# Inhibition of TGFβ Signaling Increases Direct Conversion of Fibroblasts to Induced Cardiomyocytes

**DOI:** 10.1371/journal.pone.0089678

**Published:** 2014-02-26

**Authors:** Jamie L. Ifkovits, Russell C. Addis, Jonathan A. Epstein, John D. Gearhart

**Affiliations:** Department of Cell and Developmental Biology, Institute for Regenerative Medicine, Perelman School of Medicine, University of Pennsylvania, Philadelphia, Pennsylvania, United States of America; Michigan State University, United States of America

## Abstract

Recent studies have been successful at utilizing ectopic expression of transcription factors to generate induced cardiomyocytes (iCMs) from fibroblasts, albeit at a low frequency *in vitro*. This work investigates the influence of small molecules that have been previously reported to improve differentiation to cardiomyocytes as well as reprogramming to iPSCs in conjunction with ectopic expression of the transcription factors Hand2, Nkx2.5, Gata4, Mef2C, and Tbx5 on the conversion to functional iCMs. We utilized a reporter system in which the calcium indicator GCaMP is driven by the cardiac Troponin T promoter to quantify iCM yield. The TGFβ inhibitor, SB431542 (SB), was identified as a small molecule capable of increasing the conversion of both mouse embryonic fibroblasts and adult cardiac fibroblasts to iCMs up to ∼5 fold. Further characterization revealed that inhibition of TGFβ by SB early in the reprogramming process led to the greatest increase in conversion of fibroblasts to iCMs in a dose-responsive manner. Global transcriptional analysis at Day 3 post-induction of the transcription factors revealed an increased expression of genes associated with the development of cardiac muscle in the presence of SB compared to the vehicle control. Incorporation of SB in the reprogramming process increases the efficiency of iCM generation, one of the major goals necessary to enable the use of iCMs for discovery-based applications and for the clinic.

## Introduction

Heart disease is one of the leading causes of death in the United States and around the world [1], [2]. Due to the lack of intrinsic regenerative capabilities of the adult human heart, the millions of cardiomyocytes lost due to injury are replaced with fibrotic scar tissue [3]. The recent observation that cell identity is malleable has ignited interest in the conversion of fibroblasts to induced cardiomyocytes (iCMs) as a potential strategy for the development of novel treatments for injuries to the heart. Moreover, iCMs can be utilized as tools for *in vitro* studies such as drug development and modeling of cardiac developmental disorders [3]–[14].

Successful conversion of fibroblasts to iCMs was first reported through ectopic expression of the transcription factors Gata4 (G), Mef2C (M), and Tbx5 (T) in mouse [6]. Subsequent reports have described improved conversion to iCMs with inclusion of different transcription factor combinations, including Hand2 (H) [13] as well as combinations of microRNAs [8]. Our group recently employed a genetically-encoded calcium indicator (GECI, GCaMP) driven by the cardiac Troponin T promoter to identify the transcription factor combination of HGMT plus Nkx2.5 (N), which led to enhanced iCM generation from mouse embryonic fibroblasts (MEFs) compared to the aforementioned transcription factor combinations [5]. This Troponin T-GCaMP reporter system is advantageous compared to traditional genetic reporters in that the intensity of GFP is responsive to intracellular calcium levels [15]. Since calcium oscillation links excitation to contraction in functional cardiomyocytes, this is a more stringent outcome measure of successful reprogramming to iCMs compared to traditional GFP reporters. Using this reporter system and drug-inducible transcription factor expression, our group was able to show that iCMs remain stably reprogrammed following inactivation of exogenous factors at 15 days post-induction.

Although inclusion of additional transcription factors has led to increased iCM yield compared to original reports [5], [6], [11], [13], the efficiency of conversion to iCMs remains quite low overall and is thus a hurdle for potential clinical and *in vitro* applications of iCM conversion from fibroblasts [3], [4], [16]–[19]. In the present study, we selected small molecules that have been successfully utilized to enhance differentiation of pluripotent stem and progenitor cells to cardiomyocytes [20]–[24] and reprogramming to induced pluripotent stem cells (iPSCs) [25]–[29] to investigate their impact on reprogramming to iCMs when used in conjunction with ectopic expression of HNGMT in both MEFs and cardiac fibroblasts (CFs) isolated from adult mouse hearts. We demonstrate that the TGFβ inhibitor, SB431542 (SB), increases the yield of reprogrammed iCMs by ∼5 fold compared to the vehicle control.

## Materials and Methods

### Ethics Statement

All animal work was conducted under a protocol (804335) approved by the University of Pennsylvania Institutional Animal Care and Use Committee.

### Primary Cell Isolation

Mouse embryonic fibroblasts (MEFs, isolated at E14.5) were prepared as previously described [5]. Briefly, embryos were harvested from mice of mixed background at 14.5 dpc followed by decapitation and removal of internal visceral organs, including the heart. The tissue was minced and digested with trypsin and trituration. Cells were resuspended in MEF medium (10% FBS and 2 mM L-Glutamine) and plated onto one 10 cm dish per embryo. After 24 hours, cells were passaged at 1∶3 (passage 1). MEFs were used at passages 3–5 for all reprogramming experiments.

Adult mouse cardiac fibroblasts were prepared as previously described [5]. Hearts were removed from mice (8–12 weeks in age) and minced in cold PBS. The tissue was digested in 4 mg mL^−1^ collagenase IV (Sigma) and 10 U mL^−1^ deoxyribonuclease I (Worthington Biochemical Corporation) and agitation at 37°C for 10 minutes. Samples were spun down and resuspended in TrypLE (Invitrogen) at 37°C with agitation. After 5 minutes, medium (DMEM supplemented with 15% FBS, 1% NEAA) was added and the resulting solution was plated onto gelatin coated 6-well plates. When confluent, the cells were passaged after filtration through a 40 µM filter at 1∶1 to a gelatin coated 10 cm dishes (passage 1). Cells were then passaged 1∶5 and frozen when confluent. Cardiac fibroblasts were used at passage 3 for reprogramming experiments.

### Plasmid Information

All plasmids were constructed as previously described [5] and can be found on Addgene using the following catalog numbers: Troponin T-GCaMP5-Zeo (46027), tetO-Hand2 (46028), tetO-NKX2.5 (46029), tetO-GATA4 (46030), tetO-MEF2C (46031), tetO-TBX5 (46032). Plasmids that were used from Addgene also include: FUdeltaGW-rtTA (19780), psPAX2 (12260), pMD2.G (12259), and PGK-H2B-mCherry (21217). All plasmids were amplified in STBL3 bacteria (Invitrogen) and prepared with Qiagen MidiPrep Kits.

### Lentivirus Preparation

Lentiviral vectors were packaged into Lenti-X 293T cells (Clontech) using Lipofectamine 2000 (Invitrogen) to deliver 12 µg of the lentiviral backbone plasmid, 7.7 µg psPAX2, and 4.3 µg pMD2.G in 3 mL OPTI-MEM (Invitrogen) to ∼90% confluent 10 cm plates of 293T cells with 10 mL of fresh MEF medium. Viral supernatant was collected at 24 and 48 hours post-transfection (total ∼23 mL), filtered using a 0.45 µM filter (Millipore), aliquots were prepared and frozen at −80°C until use. Viral titer was determined using Lenti-X GoStix (Clontech) and only lentiviruses with a minimum titer of 5×10^5^ IFU mL^−1^ were used for reprogramming experiments.

### Direct Conversion of Fibroblasts to iCMs

**Figure 1:**
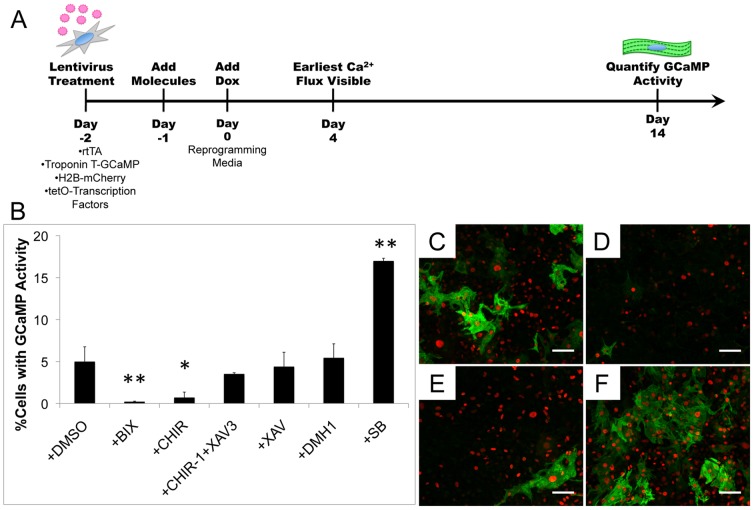
Direct conversion of mouse embryonic fibroblasts to iCMs can be influenced by treatment with small molecules. Schematic of direct reprogramming strategy of MEFs and timeline (A). Percentage of total cells with flashing Troponin T-GCaMP activity for control and small molecule treated MEFs (B). Representative immunocytochemistry images for HNGMT +DMSO (C), or +BIX (D), +CHIR+XAV (E), and +SB (F) treatments for cardiac Troponin T (green) and nuclei (red). * indicates p<0.05 and ** indicates p<0.01 compared to +DMSO control. Scale bar is 100 µM.

Direct conversion of MEFs and cardiac fibroblasts was completed using a protocol similar to that previously described [5], as shown in Figure 1A. Briefly, glass bottom 12-well plates (MatTek) were coated with poly-L-Lysine solution overnight followed by incubation with MEF medium for 1 hour prior to seeding. At Day -2 cells were dissociated using TrypLE and plated at 30k (∼7.5 k cm^−2^) per well with 250 µL of each of the FUdeltaGW-rtTA and Troponin T-GCaMP5-ZEO reporter lentivirus as well as 500 µL MEF medium (1 mL total per well). On Day-1, the culture medium was replaced with 250 µL MEF medium and 250 µL of each tetO-transcription factor lentivirus (1.5 mL total) as well as any candidate small molecules or vehicle controls (see Table 1) where indicated. After another 24 hours (Day 0), the media was changed to Reprogramming medium with doxycycline (2 µg mL^−1^) and the PGK-H2B-mCherry lentivirus. Reprogramming Medium consists of AGM (Lonza, CC-3186) without EGF and supplemented with 2 µg mL^−1^ doxycycline (Sigma). Cells were also transduced with 200 µL of PGK-H2B-mCherry lentivirus for constitutive expression of nuclear-localized mCherry expression. Reprogramming medium was changed every 2–3 days. For microarray analysis, cells were plated into standard tissue-culture treated 12 well plates (BD Falcon) using the same volumes and protocol described above. For immunocytochemistry, the identical protocol was followed, except cells were plated onto 12 mm diameter poly-L-Lysine coated glass coverslips in 24 well plates (1 coverslip per well) and using half of the volumes described above for all steps.

**Table 1:** The small molecules and growth factors that were tested for direct reprogramming of fibroblasts to iCMs.

Molecule	Manufacturer (Catalog Number)	Concentration (µg mL^−1^)	Day Added
DMSO	Sigma (D2650)	Vehicle Control	Day -1
BIX01294 (hydrochloride hydrate)	Cayman Chemical (13124)	4.8	Day -1
CHIR99021	Cayman Chemical (13122)	5.6	Day -1
XAV939	Cayman Chemical (13596)	0.781	Day -1, Day 3
SB431542	Cayman Chemical (13031)	0.048-3.84	Day -1, Day 3, or Day 6
LY364947	Cayman Chemical (13341)	0.68	Day -1
DMH1	Sigma (D8946)	0.19	Day -1
TGFβ1	R&D Systems (240-B-002)	0.002	Day -1
TGFβ2	R&D Systems (302-B2-002)	0.002	Day -1
Activin A	R&D Systems (338-AC-010)	0.010	Day -1

### Live Imaging and quantification of functional iCMs

Imaging was performed on an Olympus iX-81 microscope with Metamorph software and movies were obtained using HyperCam software (Hyperionics Technology) as previously described [5]. Briefly, for imaging TNNT2-GCaMP5 and mCherry imaging, medium in the glass-bottom plates was replaced with Tyrode's Salt Solution (Sigma) and movies were made of 10–12 unique fields within a single well of the 12 well plate using a 10× Apo objective. After a 10 sec movie collection using the GFP filter to view GCaMP, a brief segment of video was recorded with the mCherry filter to visualize all nuclei. For quantification, videos were imported into ImageJ and GCaMP+ cells were counted using the Cell Counter plug-in. For counting nuclei, one frame recorded using the mCherry filter was duplicated. After applying a threshold to the image, particle analysis was conducted on the resulting image to give the total number of nuclei present in the respective field.

### Immunocytochemistry

Samples were fixed using 4% PFA with 0.25% TritonX-100. The following primary antibodies were used: mouse anti-cardiac Troponin T (Thermo MS-295-P, 1∶200); mouse anti-myosin heavy chain (clone MF20, eBioscience 53-6503-82, 1∶200); mouse anti-sarcomeric α-actinin (clone EA53, Sigma A7811, 1∶200); rabbit anti-Isl1 (Abcam ab20670, 1∶100), rabbit anti-smooth muscle myosin heavy chain (Abcam ab53219, 1∶250), goat anti-Nkx2.5 (Santa Cruz Biotechnology sc-8687, 1∶100), chicken anti-vimentin (Millipore AB5733, 1∶500), mouse anti-Myl7 (Abcam ab68086, 1∶100), rabbit anti-Myl2 (Proteintech 10906-1-AP, 1∶100), rabbit anti-Ki-67 (Novus NB110-89719, 1∶100), and mouse anti-V5 (Invitrogen 46-0707, 1∶200). For Oil Red O staining, samples were incubated with isopropanol for 5 minutes, followed by a solution consisting of 3 parts Oil Red O solution (0.3% Oil Red O in 99% Isopropanol, Sigma) and 2 parts DI water for 5 minutes. After washing with water samples were stained with hematoxylin (Sigma), washed with water, and visualized.

### Microarray

Samples for microarray analysis were collected in Trizol at Day 3 post-transduction and RNA was prepared by the University of Pennsylvania Molecular Profiling Facility. Mouse genome-wide expression analysis was assayed using the Affymetrix Mouse Gene 1.0 ST Array and analysis was performed using the Affymetrix Microarray Suite 5.0 and PartekGS Software. Pathway and functional analyses were conducted using Ingenuity Pathway Analysis software applying a 1.5 fold change and 5% false discovery rate for MEFs and a 1.25 fold change and 5% false discovery rate for CFs. The complete microarray dataset has been deposited in the GEO database (Accession # GSE54022). Expression levels of selected genes were validated using quantitative RT-PCR.

### Quantitative RT-PCR

RNA was collected at the indicated time point using the RNeasy Mini Kit (Qiagen) and cDNA synthesis was performed using QuantiTect Reverse Transcription Kit (Qiagen). Quantitative real-time PCR was performed on an ABI 7900HT system (Applied Biosystems) using the following TaqMan gene expression assays: Gapdh (Mm99999915_g1), Hand2 (Mm00439247_m1), NKX2.5 (Hs00231763_m1), GATA4 (Hs00171403_m1), MEF2C (Hs00231149_m1), TBX5 (Hs00361155_m1), Adamtsl2 (Mm01326794_m1), Bmp2 (Mm01340178_m1), Myh6 (Mm00440359_m1), Myocd (Mm00455051_m1), Pln (Mm01201431_m1), Ppargc1a (Mm01208835_m1), and Tgfbr3 (Mm00803538_m1).

### Statistical Analysis

Data is presented as mean ± standard deviation. All statistical analysis was conducted in the JMP10 software program using a one-way ANOVA with Tukey's post-hoc testing unless otherwise noted. For all comparisons, p<0.05 was considered to be statistically significant.

## Results

### Candidate Small Molecule Screen in Mouse Embryonic Fibroblasts Conversions

The direct conversion of fibroblasts to iCMs in the presence of candidate small molecules was evaluated in MEFs utilizing inducible expression of the transcription factors HNGMT (Figure 1A) [5]. We selected five candidate small molecules from the literature based on reported abilities to influence directed differentiation from stem or progenitor cells to cardiomyocytes or reprogramming to iPSCs [20]–[24], [26], [27], [29], [30]. Specifically, the G9a histone methyltransferase inhibitor BIX01294 (BIX) was selected based on its use in differentiation of stem cells isolated from bone marrow towards cardiomyocytes [23] as well as its use in generating iPSCs [27]. The Wnt signaling pathway has been shown to play an important and biphasic role in cardiac development [31]; therefore, we selected CHIR99021 (CHIR), as an agonist of canonical Wnt signaling. CHIR has been shown to improve differentiation of human pluripotent stem cells to cardiomyocytes [22] as well as direct conversion of human fibroblasts to neurons [30]. The canonical Wnt inhibitor XAV939 (XAV) that been shown to facilitate differentiation of cardiac progenitor cells to cardiomyocytes was also tested [24]. We included an additional condition to mimic the biphasic influence of the Wnt signaling pathway in cardiogenesis [31] by switching from early administration of Wnt activation with CHIR to Wnt inhibition with XAV (CHIR-1+XAV3). Finally, since members of the TGFβ superfamily have been shown to play roles in cardiomyocyte differentiation, small molecules inhibiting different components of the pathway were evaluated. The small molecule DMH1, a BMP inhibitor, and SB431542 (SB), an Activin, Nodal, and TGFβ inhibitor, both of which have been recently shown to increase mouse embryonic stem cell differentiation to cardiomyocytes were tested [20], [21]. Moreover, SB has also been shown to increase the conversion of fibroblasts to iPSCs [26] and neurons [30].

The small molecules were added prior to inducing HNGMT expression and maintained in culture throughout the duration of the experiment, with the exception of the aforementioned CHIR-1+XAV3 group, which was transferred from CHIR to XAV treatment on Day 3. Doxycycline (Dox) was added at Day 0 to induce transcription factor expression along with a PGK-H2B-mCherry lentivirus, to produce expression of a fluorescent nuclear mCherry signal to allow for quantification of total cell number.

Upon quantification at Day 14, the baseline condition of HNGMT+DMSO generated 4.96±1.80% iCMs (Figure 1B). Importantly, there was no Troponin T-GCaMP activity observed for cells not treated with HNGMT lentiviruses at any time point. A decrease in iCM yield as well as total cell number was observed with BIX treatment (0.19±0.09%) (Figure 1B). A decrease in iCM generation was also observed for CHIR treatment (0.69±0.68%); however, addition of XAV to the media at Day 3 increased the iCM yield to 3.49±0.17% (Figure 1B). Treatment with XAV alone resulted in 4.36±1.75%, similar to the DMSO vehicle control condition (Figure 1B). A significant increase of ∼3.5 fold in iCM yield as measured by Troponin T-GCaMP was observed for cells treated with 0.5 µM SB (16.95±0.35%, Figure 1B). Moreover, addition of 2 µM SB resulted in an increase of ∼5 fold in the number of converted iCMs with Troponin T-GCaMP observed at Day 14 post-induction compared to the DMSO vehicle control.

Immunostaining at Day 14 revealed cells positive for cardiac Troponin T expression in all conditions evaluated and an increase in cells with cardiac Troponin T expression was qualitatively observed with SB treatment (Figure 1C–F). In addition, iCMs expressed multiple cardiomyocyte-specific markers for both DMSO and SB treatment groups, including α-actinin, α-myosin heavy chain, myosin light chain 2, and myosin light chain 7 (Figure S1). Robust beating of cells was seen as early as 11 days post-induction (Supporting Information Movie S1). Moreover, groups of cells beating in unison were also observed (Supporting Information Movie S2).

Therefore, we concluded that treatment with the small molecule SB in conjunction with ectopic expression of the transcription factors HNGMT increases the conversion of MEFs to iCMs compared to the DMSO control. Since SB is a well established and specific inhibitor of the Alk4, Alk5, and Alk7 components of the TGFβ signaling pathway [32], we sought to characterize the specific members of this pathway that are modulated by SB and may play a role in conversion to iCMs. We also investigated if the increased number of iCMs may be due to proliferation of nascent converted cells or apoptosis of the initial cell population, as TGFβ is known to influence these cellular processes [33]–[35].

### Inhibition of TGFβ Increases the Efficiency of iCM Generation and is Time Dependent

SB is a small molecule inhibitor of the Activin, Nodal, and TGFβ components of the TGFβ superfamily signaling pathway. In order to investigate the specificity of inhibition on the resulting iCM yield, LY364947 (LY), an inhibitor of the TGFβ but not Activin and Nodal components [36] was added on Day -1 according to the aforementioned protocol. There was a significant increase in the number of cells with Troponin T-GCaMP activity for samples treated with SB or LY compared to the DMSO vehicle control (Figure S2A). Interestingly, inhibition of TGFβ with SB or LY was also associated with differentiation of adipocytes in the culture (Figure S2B), as has been reported [37]. Therefore, inhibition of TGFβ is most likely to be responsible for the increase in iCM yield associated with SB or LY treatment as opposed to inhibition of the Activin or Nodal family members.

**Figure 2:**
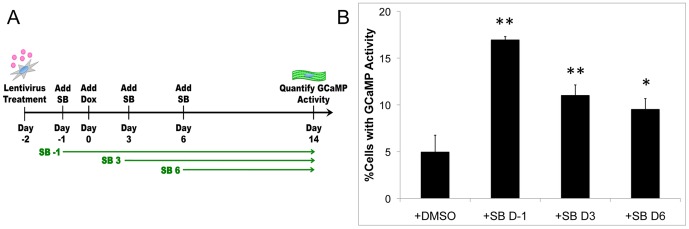
Inhibition of TGFβ early in the conversion process leads to the greatest increase in iCM yield. Schematic depicting the addition of SB at different time points following induction of the transcription factors (A). Quantification at Day 14 of the number of cells with Troponin T-GCaMP activity upon addition of SB at Day -1 (D-1), 3 (D3), and 6 (D6) (B). * indicates p<0.05 and ** indicates p<0.01 compared to +DMSO control.

In order to gain further insight into the influence of SB treatment administration on conversion to iCMs, SB was added to the reprogramming scheme on Day -1, Day 3, or Day 6 and maintained in the culture media throughout the experiment as outlined in Figure 2A. Although an increase in cells with Troponin T-GCaMP activity was observed regardless of the timing of SB addition, the greatest increase in iCM yield was observed upon addition at Day -1 of conversion (Figure 2B). Thus, the greatest benefit of TGFβ inhibition appears to occur simultaneously with exogenous transcription factor expression. Based on these results, SB was added at Day -1 for all subsequent experiments.

### SB Does Not Affect Transduction Efficiency or Cellular Proliferation

**Figure 3:**
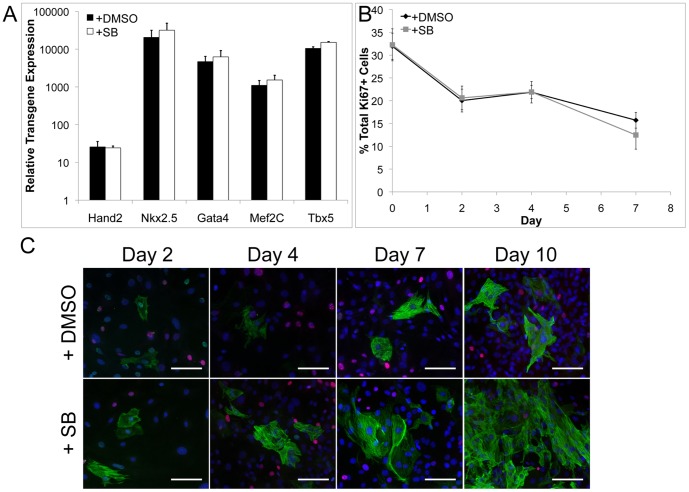
The increase in iCM number with SB treatment is not due to increased transgene expression or changes in cell proliferation. Evaluation of gene expression of the transgenes at Day 2 via qPCR is not significantly different between +DMSO (black) control and +SB treatment groups (white, A). Evaluation of overall cell proliferation with Ki67 staining in MEFs with time for +DMSO (black) and +SB treatment groups (grey) (B). A time course evaluation of Troponin T (green) and Ki67 (red) did any dual labeled cells for either the +DMSO (top row) and +SB (bottom row) treated groups (C). Scale bar is 100 µM.

We considered several possible mechanisms in which SB may lead to an increased yield of iCMs. The influence of SB treatment on viral transduction efficiency was evaluated by utilizing qPCR to evaluate transgene expression at Day 2 post-induction in the presence DMSO or SB. No difference in transgene expression between the two groups was observed for any of the transcription factors utilized to convert MEFs to iCMs (Figure 3A). Moreover, viral transduction efficiency was also evaluated via immunostaining for the V5 epitope tag that is present at the C-terminus of the delivered transcription factors. Specifically, MEFs were transduced with only the tetO-Nkx2.5-V5 lentivirus in the presence of DMSO or SB and stained for V5 expression at Day 2 post-induction. Again, no significant difference in V5 expression was observed (DMSO 73.2±2.7% and SB 68.4±2.5%, Figure S3A).

We next examined the possibility that SB may be affecting proliferation of iCMs or reducing proliferation and/or inducing apoptosis of non-converted fibroblasts. A time course of immunostaining for cardiac Troponin T and the proliferation marker Ki67 was conducted to investigate overall cellular proliferation as well as proliferation of iCMs for cells treated with the DMSO vehicle control or SB. No difference in overall cellular proliferation was observed between the DMSO and SB treatment groups at any time point (Figure 3B). As shown in Figure 3C, cardiac Troponin T positive cells are evident as early as Day 2, however the staining is faint and organized sarcomeres are not present at this time point. Although the number of cells and intensity of staining increases with time, co-expression of cardiac Troponin T and Ki67 was not observed for either condition at any time point (Figure 3C).

Since the TGFβ signaling pathway is also known to influence cellular apoptosis [35], a time course for TUNEL staining was also conducted. Overall, there were very few TUNEL positive apoptotic cells at each time point and no difference between the DMSO vehicle control and SB treatment groups was observed (Figure S3B). Therefore, based on these results as well as our previous observations, the increased percentage of iCMs observed with SB treatment suggests that inhibition of TGFβ plays an important role in the early events involved in reprogramming as opposed to inducing proliferation of iCMs or apoptosis of non-reprogrammed MEFs.

### Conversion of Adult Mouse Cardiac Fibroblasts to iCMs is Increased by Inhibition of TGFβ

Adult cardiac fibroblasts are an attractive target for the development of direct *in vivo* conversion strategies, as has been demonstrated by other groups [7], [12], [13]. The targeted delivery of small molecules and growth factors to the heart after injury has also been demonstrated [38], suggesting that delivery in conjunction with transcription factors for *in vivo* conversion is possible. Therefore, we investigated if inhibition of TGFβ with SB treatment would also increase the conversion of adult cardiac fibroblasts to iCMs as quantified by Troponin T-GCaMP activity.

**Figure 4:**
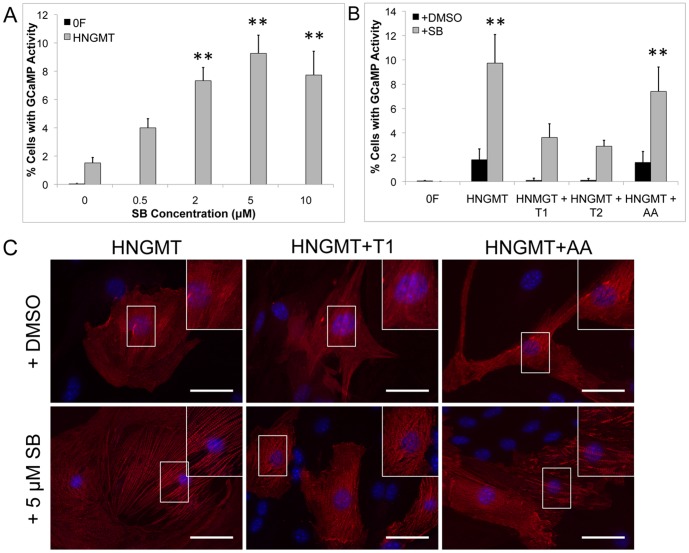
Direct conversion of adult mouse cardiac fibroblasts (CFs) to iCMs. Conversion efficiency to iCMs increases in yield with increasing concentration of SB (A). Direct conversion of CFs to iCMs is reduced with addition of TGFβ1 (black, HNGMT+T1) and TGFβ2 (black, HNGMT+T2) to the culture medium, but not Activin A (black, HNGMT+AA). Addition of SB (grey) increases the yield of iCMs generated in the presence of TGFβ1 (grey, HNGMT+T1) and TGFβ2 (grey, HNGMT+T2 and Activin A (grey, HNGMT+AA) (B). Immunostaining for αActinin (red) and DAPI (blue) demonstrate increased sarcomeric structure with SB treatment for HNGMT+T1 (C). ** indicates p<0.01 compared to HNGMT+DMSO control.

The cardiac fibroblasts were isolated from adult mice and did not express markers of cardiac progenitor cells or cardiomyocytes upon immunostaining (Figure S4). Immunocytochemistry conducted at Day 0 revealed that 99.47±0.14% of the initial cell population was positive for vimentin, an intermediate filament that is commonly used to identify cardiac fibroblasts [6]. Conversion of adult cardiac fibroblasts to iCMs was evaluated in the presence of DMSO as well as varying concentrations of SB (0.5, 2, 5, and 10 µM). Quantification of Troponin T-GCaMP activity at Day 14 post-induction revealed a conversion of 1.52±0.38% for the DMSO vehicle control condition (Figure 4A). The number of cells with Troponin T-GCaMP activity increased with increasing SB concentration and reached a plateau of 9.27±1.28% for treatment with 5 µM SB (Figure 4A). Importantly, inhibition of TGFβ signaling in the cardiac fibroblasts did not lead to differentiation of adipocytes as was observed in the MEFs, therefore confirming the increase in iCM yield is due to the presence of SB as opposed to possible paracrine effects of adipocytes.

Converted iCMs displayed expression of several cardiomyocyte markers via immunostaining, such as cardiac Troponin T (Figure S5A), α-actinin and α-myosin heavy chain (Figure S5B), and myosin light chain 2 and myosin light chain 7 (Figure S5C). Beating of iCMs derived from adult cardiac fibroblasts was evident as early as Day 16 post-induction (Supporting Information Movie S3) and was maintained up to at least Day 34 in culture (Supporting Information Movie S4).

### Conversion of Fibroblasts to iCMs is Suppressed in the Presence of TGFβ Ligands But Can be Rescued by Addition of SB

We next investigated the effect of the presence of the TGFβ signaling ligands, TGFβ1 and TGFβ2 on conversion of adult mouse cardiac fibroblasts to iCMs. Activin A was also included to confirm the specificity of the response to the TGFβ ligands. As with the small molecules, the growth factors were added to the cultured cells at Day -1 of the experimental scheme. Almost no cells with Troponin T-GCaMP activity were evident on Day 14 upon addition of TGFβ1 (0.098±0.18%) or TGFβ2 (0.106±0.15%) to the culture compared to the DMSO vehicle control (1.79±0.89%, Figure 4B) and Activin A (1.57±0.90%, Figure 4B). Although occasional cardiac Troponin T positive cells were visualized upon immunostaining at Day 14 post-induction in the TGFβ1 or TGFβ2 treatment groups (Figure 4C), reduced staining intensity and sarcomeric organization were observed compared to the DMSO control group. Similar observations were made upon evaluation in MEFs, where a 98% or 96% reduction in the number of cells with Troponin T-GCaMP activity in the presence of TGFβ1 or TGFβ2, respectively was observed.

We further evaluated if the addition of SB could rescue the conversion of fibroblasts to iCMs in the presence of TGFβ1 or TGFβ2. As previously observed, addition of 5 µM SB led to an increase of ∼5.5 fold in the number of cells with Troponin T-GCaMP activity at Day 14 for the no growth factor treatment (9.74±2.34%, Figure 4B) and Activin A (7.41±2.00%, Figure 4B) treatment groups compared to the DMSO vehicle control (1.79±0.89%, Figure 4B). Addition of 5 µM SB at Day -1 in conjunction with TGFβ1 or TGFβ2 led to a significant increase to 3.16±1.13% or 2.90±0.5% (Figure 4B), respectively, compared to treatment with the growth factors alone. Furthermore, addition of 10 µM SB in conjunction with TGFβ1 led to a greater increase in the percentage of cells with Troponin T-GCaMP activity to 4.65±1.78%. Qualitatively, an increase in the number of cardiac Troponin T positive cells visualized by immunostaining was also observed with the organization of sarcomeres appearing similar to that of the DMSO vehicle control group (Figure 4C). Although there was an increase compared to the DMSO vehicle control group, addition of SB to the TGFβ treatments did not rescue the response to the level of the SB alone treatment group.

### Inhibition of TGFβ with SB Leads to Enhanced Early Expression of Cardiac Genes

In an attempt to identify the mode of action in which inhibition of TGFβ with SB leads to increased conversion of fibroblasts to iCMs, global transcriptional analysis of cells cultured in the presence of the DMSO vehicle control or SB was conducted. Based on our observation that early administration of SB leads to the greatest increase in conversion to iCMs, samples were isolated at Day 3 post-induction to gain insight into early differences in reprogramming caused by inhibition of TGFβ. Moreover, as adipocytes were also observed in MEFs treated with SB, cells treated with DMSO or SB without HNGMT transcription factors (0TF) were also included in our analysis.

**Figure 5:**
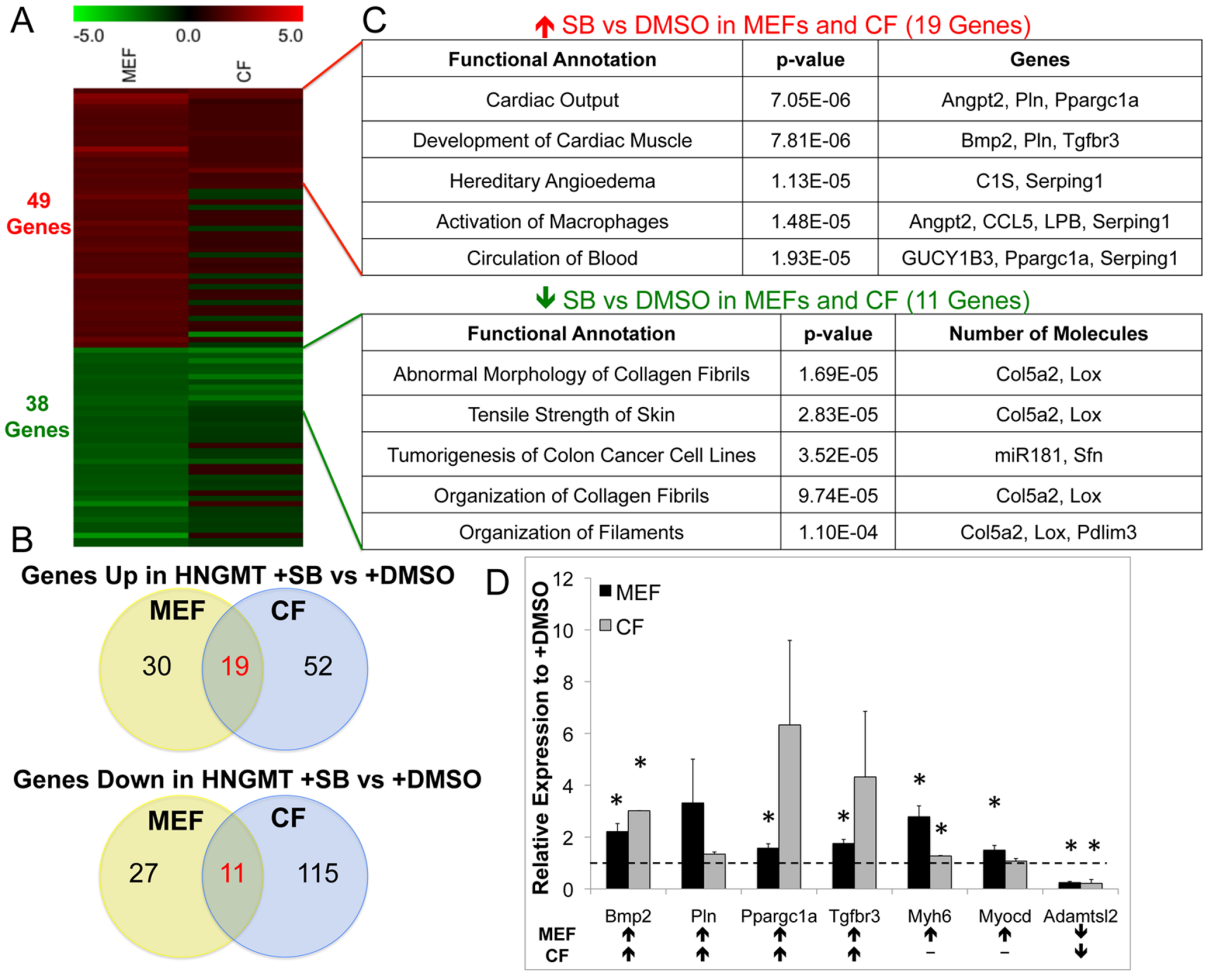
Evaluation of gene expression changes in MEFs and CFs during conversion with and without SB. Heatmap depicting the 49 and 38 genes up-regulated and down-regulated, respecively exclusively in MEFs for HNGMT + SB versus HNGMT+DMSO as well as the expression of the same genes in CFs (A). Venn diagrams demonstrating the overlap of 19 genes up-regulated HNGMT+SB for both MEFs and CFs as well as the 11 genes down-regulated in HNGMT+SB for both MEFs and CFs (B). The top five groups by p-value from as identified by functional analysis of the 19 commonly up-regulated and 11 commonly down-regulated genes in both MEFs and CFs for HNGMT+SB treatment (C). Quantitative RT-PCR results for genes identified to be involved in development of cardiac muscle and up-regulated by both MEFs and CFs (Bmp2, Pln, Ppargc1a, and Tgfbr3), or MEFs (Myh6 and Myocd), or down-regulated in both MEFs and CFs (Adamtsl2) (D). * indicates p<0.05 compared to the HNGMT+DMSO control for that gene and cell type by Students t-test.

Applying the criteria of a fold change of at least 1.5 and a false discovery rate of 5% to the dataset consisting of 23,222 probes, there were 75 genes up-regulated and 93 genes down-regulated in MEFs in the presence of SB compared to DMSO for HNGMT transduced cells. Genes commonly associated with fibroblasts (e.g. vimentin and S100a4) did not meet these criteria and thus were not different between the DMSO and SB treatment groups (Figure S6). Comparison of the HNGMT+SB and 0TF+SB groups revealed that 26 of the 75 genes were up-regulated in both groups while 49 were up-regulated exclusively in the HNGMT+SB treatment group (Figure 5A and Figure S6). Similarly, 55 of the 93 genes were down-regulated in the SB treatment groups for HNGMT and 0TF and 38 were down-regulated exclusively in the HNGMT+SB group (Figure 5A and Figure S6). Functional analysis using Ingenuity Pathway Analysis software of genes up-regulated exclusively in the HNGMT+SB group revealed enrichment for functions related to the development of the cardiovascular system and cardiac muscle (Figure S6). Genes that were down-regulated clustered by various functional groups including involvement in the inflammatory response and neoplasia (Figure S6).

Nineteen genes that were up-regulated in MEFs in HNGMT+SB were also up-regulated in adult mouse cardiac fibroblasts under the same conditions (Figure 5A, 5B). Functional analysis of these 19 genes revealed enrichment of genes associated with cardiac output and the development of cardiac muscle (Figure 5C). Similarly, 11 of the genes down-regulated in MEFs in HNGMT+SB were also down-regulated in the adult mouse cardiac fibroblasts (Figure 5A, 5B). A complete list of the 19 genes up-regulated by both cell types and the 11 genes down-regulated by both cell types is presented in Supporting Information Tables S1 and S2. The expression of several of the genes that were identified as being involved in the development of cardiac muscle for both MEFs and CFs as well as MEFs alone (Myh6 and Myocd) as indicated in the functional analysis were verified using qRT-PCR (Figure 5D). The gene Adamtsl2, which was down-regulated in both cell types upon SB treatment, was also validated by qPCR (Figure 5D).

## Discussion

The direct conversion of fibroblasts to iCMs has been demonstrated following the induced expression of different combinations of transcription factors and/or microRNAs both *in vitro* [5], [6], [8], [10], [11], [14] and *in vivo* post-infarct in mouse models [7]–[9], [12], [13]. Given the differences in initial starting cell populations, transcription factor combinations utilized for conversion, time points evaluated, and metrics used to quantify successful conversion, wide ranges of efficiencies have been reported. For example, the first demonstration of the conversion of neonatal mouse cardiac fibroblasts to iCMs utilized an αMHC-GFP reporter system to observe ∼20% of cells expressed GFP 10 days after treatment with the transcription factor combination GMT [6]. However, not all of the αMHC-GFP cells also expressed cardiac Troponin T and only a fraction displayed some degree of spontaneous calcium activity [6]. Issues of determining conversion efficiency and defining bona iCMs were recently reviewed [4].

Our group recently conducted a side-by-side comparison of reprogramming of MEFs and adult mouse CFs to iCMs using induced expression of various combinations of transcription factors (e.g., GMT, HGMT, and NGMT) reported in the literature [6], [11], [13]. We utilized a genetically encoded calcium indicator (GCaMP) driven by the cardiac Troponin T promoter as the main outcome metric of conversion to iCMs. As excitation and contraction are linked by calcium flux, this is a more functional and therefore rigorous metric of success compared to the use of traditional genetic reporter systems. At 14 days post-induction, the greatest number of iCMs was observed with the combination HNGMT (∼2%), while the combination GMT yielded <0.25% of cells with Troponin T-GCaMP activity [5]. Although many of the aforementioned studies have raised excitement about the possibility of generating new cardiomyocytes, it is clear that technical and biological hurdles remain to be overcome, including the low efficiency of iCM conversion from fibroblasts [3], [4], [16]–[19]. Here, we present work demonstrating that inhibition of the TGFβ signaling pathway with a single small molecule leads to a significant increase of at least 5 fold in the number of reprogrammed iCMs generated from MEFs as well as adult mouse cardiac fibroblasts.

Small molecules can be delivered in a site-specific manner to the heart through a variety of means, such as encapsulation within targeted nanoparticles [38], which may be attractive for *in vivo* conversion strategies. Additionally, small molecules have been utilized to enhance directed differentiation towards cardiomyocytes [20]–[24] as well as reprogramming to iPSCs [25]–[29]. Therefore, we hypothesized that some of these small molecules could be used to increase the efficiency of reprogramming to iCMs. When we tested an initial set of small molecules with the transcription factor combination of HNGMT, only the TGFβ inhibitor SB significantly increased the yield of functional iCMs, as quantified by using the Troponin T-GCaMP reporter previous developed by our group [5]. Inhibition of TGFβ with SB has also been utilized either alone or in conjunction with other small molecules to enhance reprogramming to iPSC from a variety of cell types including MEFs [26], [39], human foreskin fibroblasts [25], and skeletal myoblasts [28] as well as direct conversion to neurons [30] and endothelial cells [40].

The TGFβ superfamily is known to influence several cellular functions, including proliferation, apoptosis, and differentiation. Moreover, the TGFβ ligands are known to elicit different responses based on the cellular context [33]–[35]. The superfamily includes the TGFβ ligands, Activins, Nodal, GDFs, and BMPs, which signal through specific transmembrane receptors [33]–[35]. SB functions via specifically inhibiting the ability of the Activin and Nodal receptors, Alk4 and Alk7, and the TGFβ receptor, Alk5, to phosphorylate Smad2/Smad3 [32], which is necessary for subsequent translocation to the nucleus for initiation of downstream signaling [32].

Upon further characterization utilizing additional small molecules targeting different TGFβ superfamily receptors, we confirmed that inhibition of TGFβ component of the superfamily in the reprogramming process leads to increased iCM yield in a dose-responsive manner. There were no obvious differences in phenotype between iCMs generated with SB or the DMSO vehicle control as observed by immunocytochemistry. Additionally, HNGMT induction in the presence of the TGFβ ligands, but not Activin A, led to a significant reduction in iCM yield, further confirming the specificity of inhibition to the TGFβ ligands. Similar to work described in the iPSC field, we observed that the increase in reprogramming efficiency with SB treatment was not due to increased transgene expression, reduced apoptosis, or proliferation of iCMs [26].

In an attempt to understand how inhibition of TGFβ may lead to increased conversion to iCMs, global transcriptional analysis was conducted on both MEFs and CFs in the presence of SB and the DMSO vehicle control. Based on our observations in MEFs that early addition of SB leads to the greatest increase in iCM yield, we collected samples for transcriptional analysis at Day 3 post-induction in order to investigate early molecular changes in reprogramming. However, we acknowledge that it is possible that important early events in the conversion process may transient in nature and therefore missed at this discrete time point.

Studies in the iPSC field have suggested that inhibition of TGFβ may facilitate reprogramming to iPSC by depression of fibroblast gene expression programs [26], [39], however differences in expression of genes commonly associated with fibroblasts (e.g., S100A4) were not identified by our analysis. Differences in gene expression for canonical components of the TGFβ signaling pathway (e.g. Smad2, Smad3, and Smad4) were also not observed between the DMSO and SB treatment groups. However, this is not surprising as many of these components are constantly present but require phosphorylation for activation [33]. Interestingly, there are 11 genes based on our fold change and false discovery rate criteria that are down-regulated in both MEFs and CFs when treated with SB as opposed to DMSO, some of which are involved in collagen fiber organization as well as tumorigenesis.

A total of 49 genes were up-regulated after HNGMT treatment in MEFs between SB and DMSO groups. Functional analysis of these genes showed enhancement of genes involved in development of the cardiovascular system and morphogenesis of cardiac muscle. A subset of 19 genes was also up-regulated in CFs treated with HNGMT+SB, several of which are also associated with development of cardiac muscle (e.g., Bmp2, Pln, and Tgfbr3) and cardiac output (e.g., Ppargc1a, Pln, and Angpt2). Although several of the genes identified in the MEFs HNGMT+SB treatment group involved in the development of the cardiac muscle cluster did not meet our fold change criteria in CFs, several genes (e.g., Myh6 and Myocd) had increased expression compared to the CF HNGMT+DMSO group. Differences in the kinetics of reprogramming between the MEFs and CFs may be responsible for reduced expression of some genes in CFs compared to MEFs.

Thus, treatment with SB early in reprogramming is associated with enhanced expression of genes related to cardiac muscle development, however it is unclear if this is a direct or indirect result of TGFβ inhibition. Interestingly, a recent report describing the conversion of fibroblasts derived from human embryonic stem cells demonstrated that inhibition of TGFβ led to a reduction in the percentage of reprogrammed iCMs expressing an αMHC-mCherry reporter [41]. These results may reflect a species difference in addition to differences in transcription factors utilized for conversion. Given the complex nature of the TGFβ signaling pathway, it is possible that multiple, interacting targets and pathways may be involved in the mechanism behind the enhanced conversion of iCMs with SB treatment and induction of HNGMT. Phosphorylated Smad complexes regulate gene expression by binding to DNA with partner transcription factors, which subsequently activate or repress transcription through recruitment of chromatin modifying co-activators (e.g., HATs) or co-repressors (e.g., HDACs) [33]–[35], [42]. For example, it is possible that inhibition of TGFβ may lead to inhibition of a gene that represses transcription of a second gene, as is the case in epithelial-to-mesenchymal transition for the genes Atf3 and Id1 [35].

In conclusion, we have shown that inhibition of TGFβ signaling with a single small molecule leads to a significant enhancement in the number of iCMs generated from two different initial cell populations. To the best of our knowledge, this work demonstrates the highest efficiency of direct conversion to iCMs. Overcoming the technological and biological limitations of direct conversion allows for practical execution of this exciting technology for the development of novel treatment strategies as well as drug development and discovery-based applications.

## Supporting Information

Figure S1 **Immunocytochemistry staining for iCMs generated from MEFs at Day 14 post-induction.** Scale bar is 50 µM.
(TIF)

Figure S2 **Inhibition of TGFβ signaling ligands in MEFs leads to increased iCM yield.** Troponin T-GCaMP analysis at Day 14 post-induction with DMSO vehicle control, LY364947 (LY) that inhibits TGFβ alone, and SB that inhibits TGFβ and Activin/Nodal components of the superfamily (A). Oil Red O staining for adipocytes also present in cultures of iCMs generated from MEFs and treated with the different small molecules at Day 14 post-induction. Scale bar is 200 µM.
(TIF)

Figure S3 **Evaluation of Nkx2.5-V5 transgene expression (green) at Day 2 post-induction between DMSO and SB treatment groups (A).** Time course staining for TUNEL (green) and DAPI (blue) for iCMs derived from MEFs at Day 2, Day 4, and Day 7 post-induction for +DMSO (top row) and +SB (bottom row) (B). Scale bar is 100 µM.
(TIF)

Figure S4 **Characterization of starting adult mouse cardiac fibroblasts by immunocytochemistry staining for Nkx2.5 (green, A), vimentin (red, B–E), Isl1 (green, B), α-actinin (green, C), α-myosin heavy chain (green, D), and cardiac troponin (green, E).** As expected, CFs stain positively for vimenin but negative for markers of cardiomyocytes and cardiac progenitor cells. Scale bar is 100 µM.
(TIF)

Figure S5 **Representative immunocytochemistry at Day 14 of iCMs for +DMSO (top row) and + 5** µ**M SB treatment (bottom row) for the indicated proteins.** Scale bar is 100 µM (B–C) or 50 µM (D).
(TIF)

Figure S6 **Heatmap for genes commonly associated with fibroblasts for MEFs and CFs at Day 3 post-induction in HNGMT+SB versus HNGMT+DMSO, with fold change and false discovery rate (FDR) values on the table to the right (top).** Heatmap of genes up-regulated and down-regulated in MEFs for HNGMT+SB versus HNGMT+DMSO with functional annotations for the genes up-regulated or down-regulated exclusively in HNGMT+SB versus HNGMT+DMSO on the tables to the right (bottom).
(TIF)

Movie S1 **Robust beating of iCMs converted from MEFs is evident as early as Day 11 post-induction.**
(MP4)

Movie S2 **Groups of iCMs converted from MEFs beating synchronously at Day 18 post-induction.**
(MP4)

Movie S3 **Beating of iCMs converted from adult mouse CFs was observed as early as Day 16 post-induction.**
(MP4)

Movie S4 **iCMs directly reprogrammed from adult mouse CFs were observed to be beating through at least 34 days in culture post-induction.**
(MP4)

Table S1 **Genes up-regulated in HNGMT+SB versus HNGMT+DMSO for both MEFs and CFs at Day 3 post-induction.**
(DOCX)

Table S2 **Genes that are down-regulated in HNGMT+SB versus HNGMT+DMSO for both MEFs and CFs at Day 3 post-induction.**
(DOCX)
