# Supplementary figures and images for: Inhibition of TGFβ Signaling Increases Direct Conversion of Fibroblasts to Induced Cardiomyocytes
Source: PLoS One. 2014 Feb 26;9(2):e89678. doi: 10.1371/journal.pone.0089678 (PMC3935923; doi:10.1371/journal.pone.0089678)

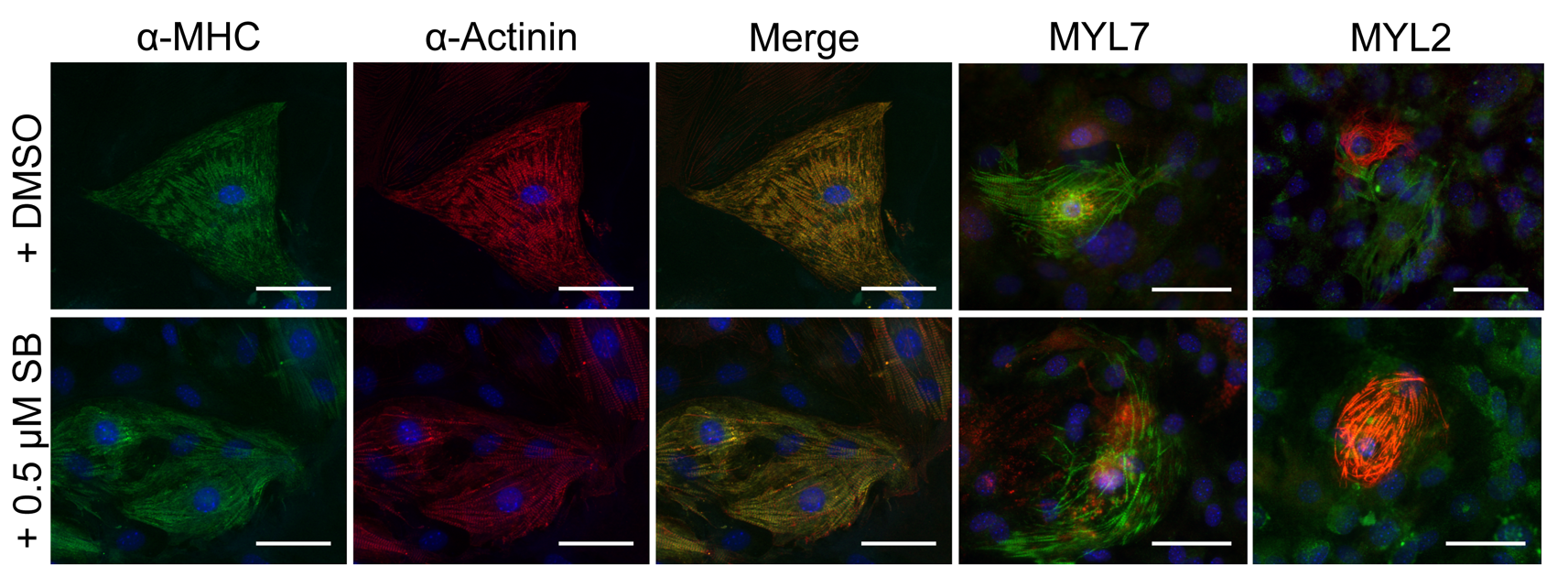

Supplement: Figure S1 — Immunocytochemistry staining for iCMs generated from MEFs at Day 14 post-induction. Scale bar is 50 µM. (TIF) [file pone.0089678.s001.tif]

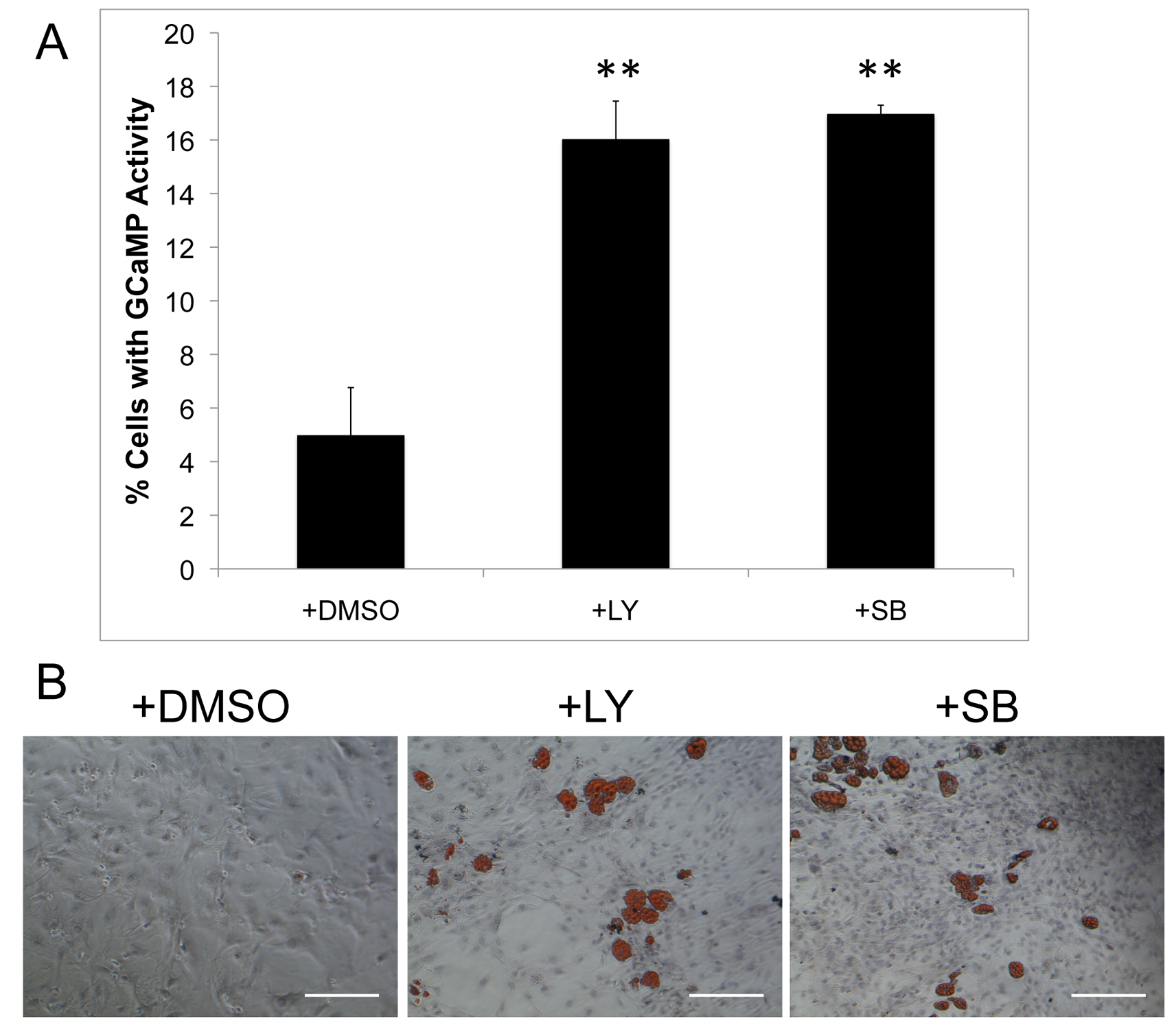

Supplement: Figure S2 — Inhibition of TGFβ signaling ligands in MEFs leads to increased iCM yield. Troponin T-GCaMP analysis at Day 14 post-induction with DMSO vehicle control, LY364947 (LY) that inhibits TGFβ alone, and SB that inhibits TGFβ and Activin/Nodal components of the superfamily (A). Oil Red O staining for adipocytes also present in cultures of iCMs generated from MEFs and treated with the different small molecules at Day 14 post-induction. Scale bar is 200 µM. (TIF) [file pone.0089678.s002.tif]

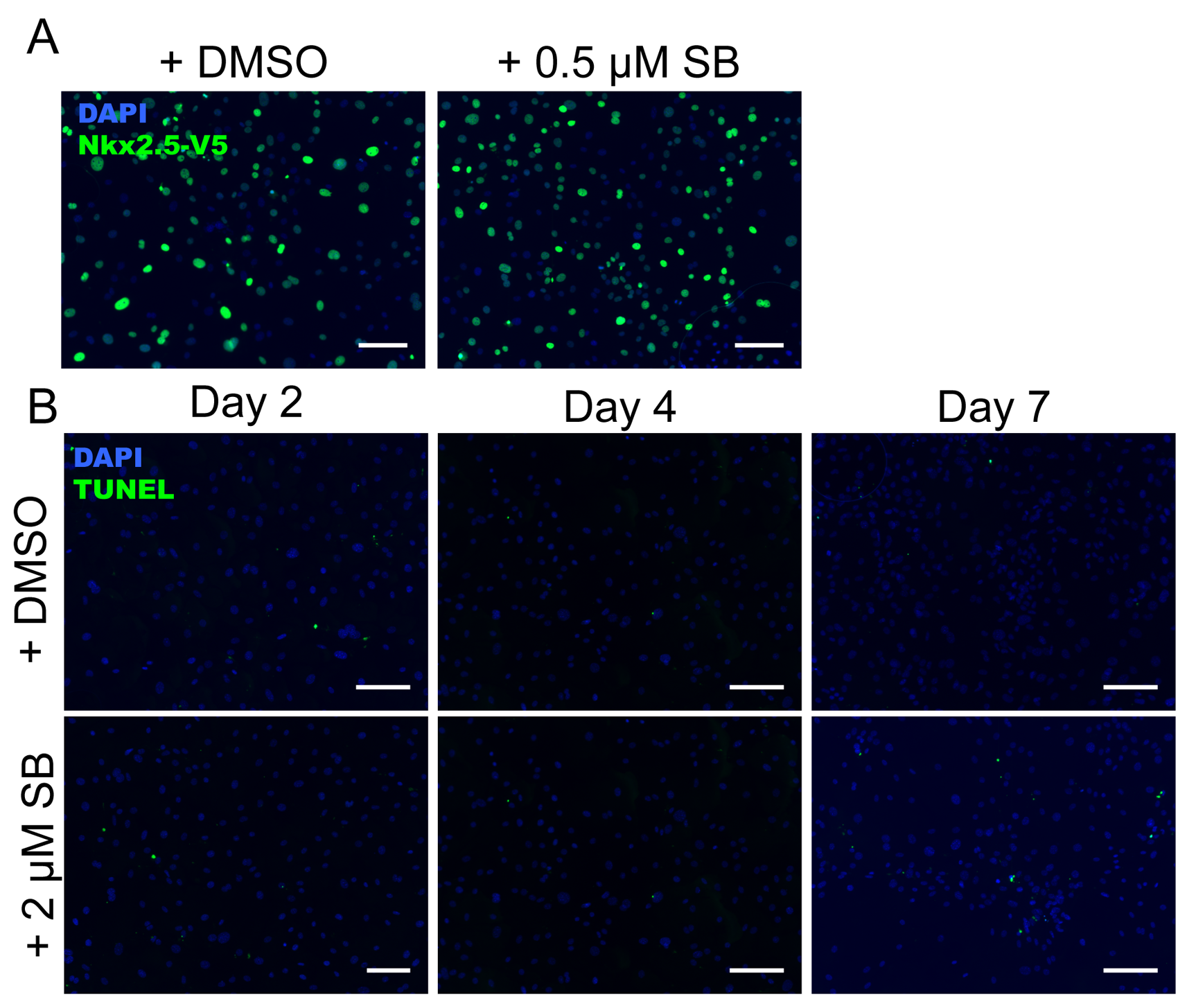

Supplement: Figure S3 — Evaluation of Nkx2.5-V5 transgene expression (green) at Day 2 post-induction between DMSO and SB treatment groups (A). Time course staining for TUNEL (green) and DAPI (blue) for iCMs derived from MEFs at Day 2, Day 4, and Day 7 post-induction for +DMSO (top row) and +SB (bottom row) (B). Scale bar is 100 µM. (TIF) [file pone.0089678.s003.tif]

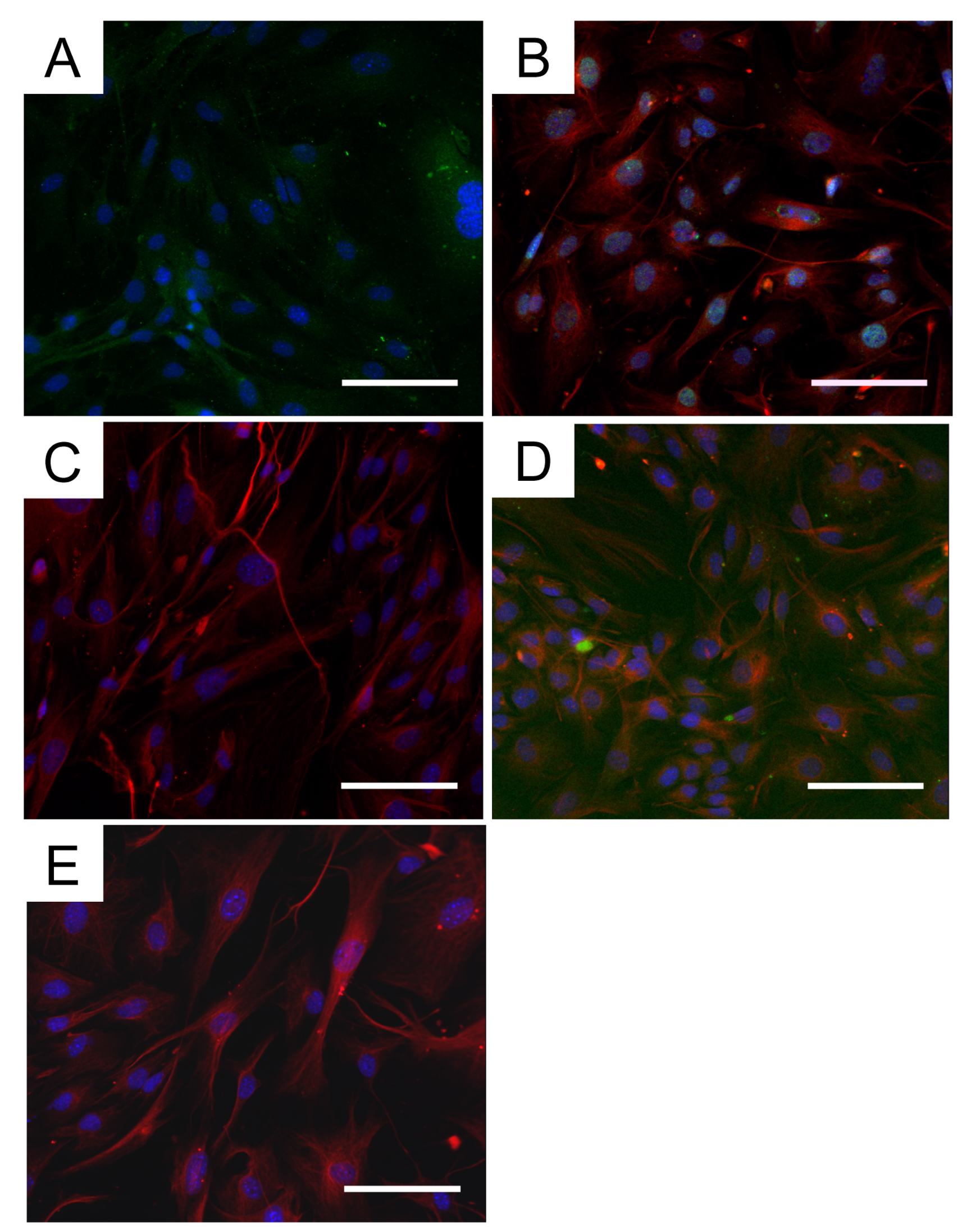

Supplement: Figure S4 — Characterization of starting adult mouse cardiac fibroblasts by immunocytochemistry staining for Nkx2.5 (green, A), vimentin (red, B–E), Isl1 (green, B), α-actinin (green, C), α-myosin heavy chain (green, D), and cardiac troponin (green, E). As expected, CFs stain positively for vimenin but negative for markers of cardiomyocytes and cardiac progenitor cells. Scale bar is 100 µM. (TIF) [file pone.0089678.s004.tif]

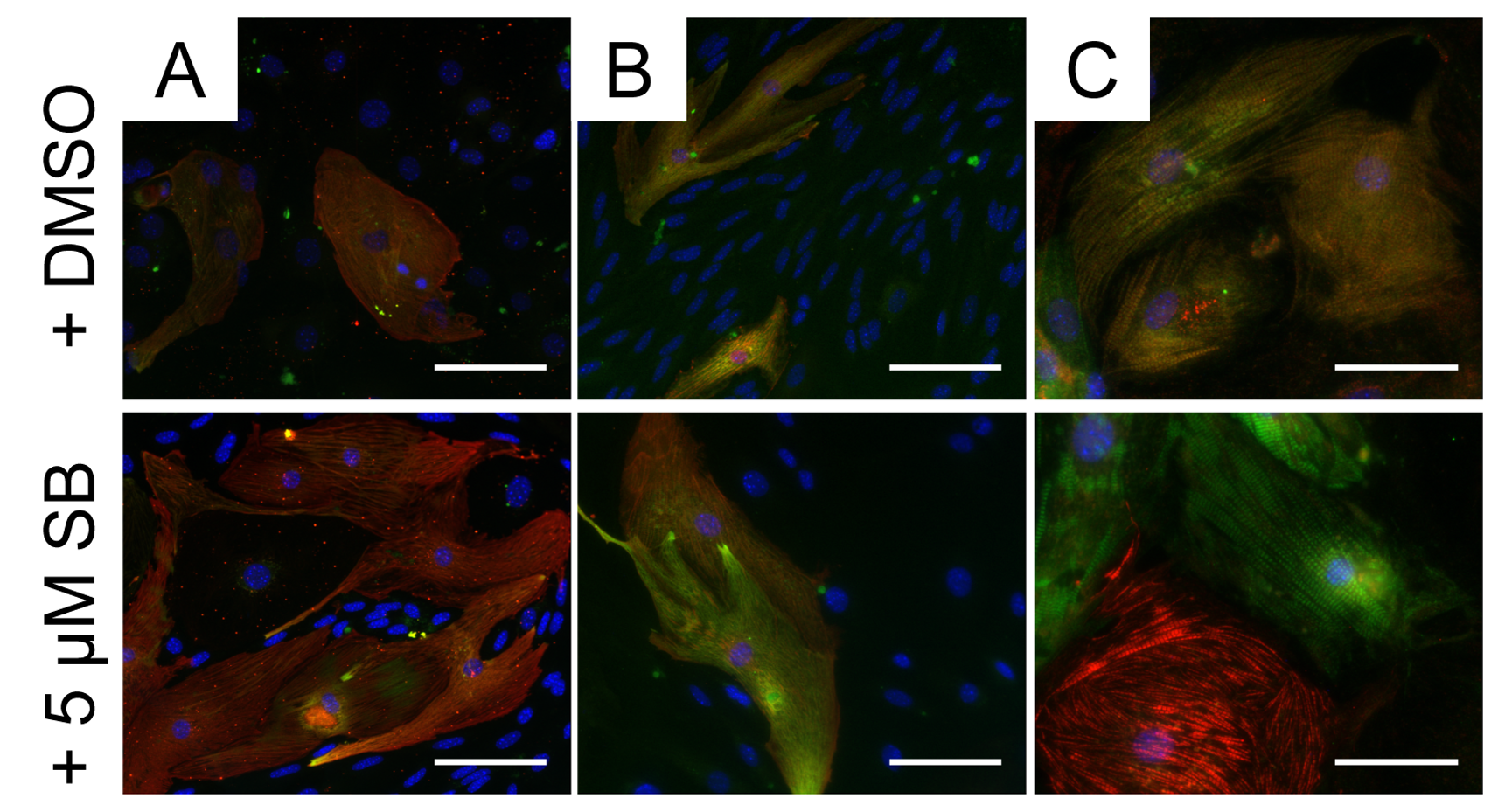

Supplement: Figure S5 — Representative immunocytochemistry at Day 14 of iCMs for +DMSO (top row) and + 5 µM SB treatment (bottom row) for the indicated proteins. Scale bar is 100 µM (B–C) or 50 µM (D). (TIF) [file pone.0089678.s005.tif]

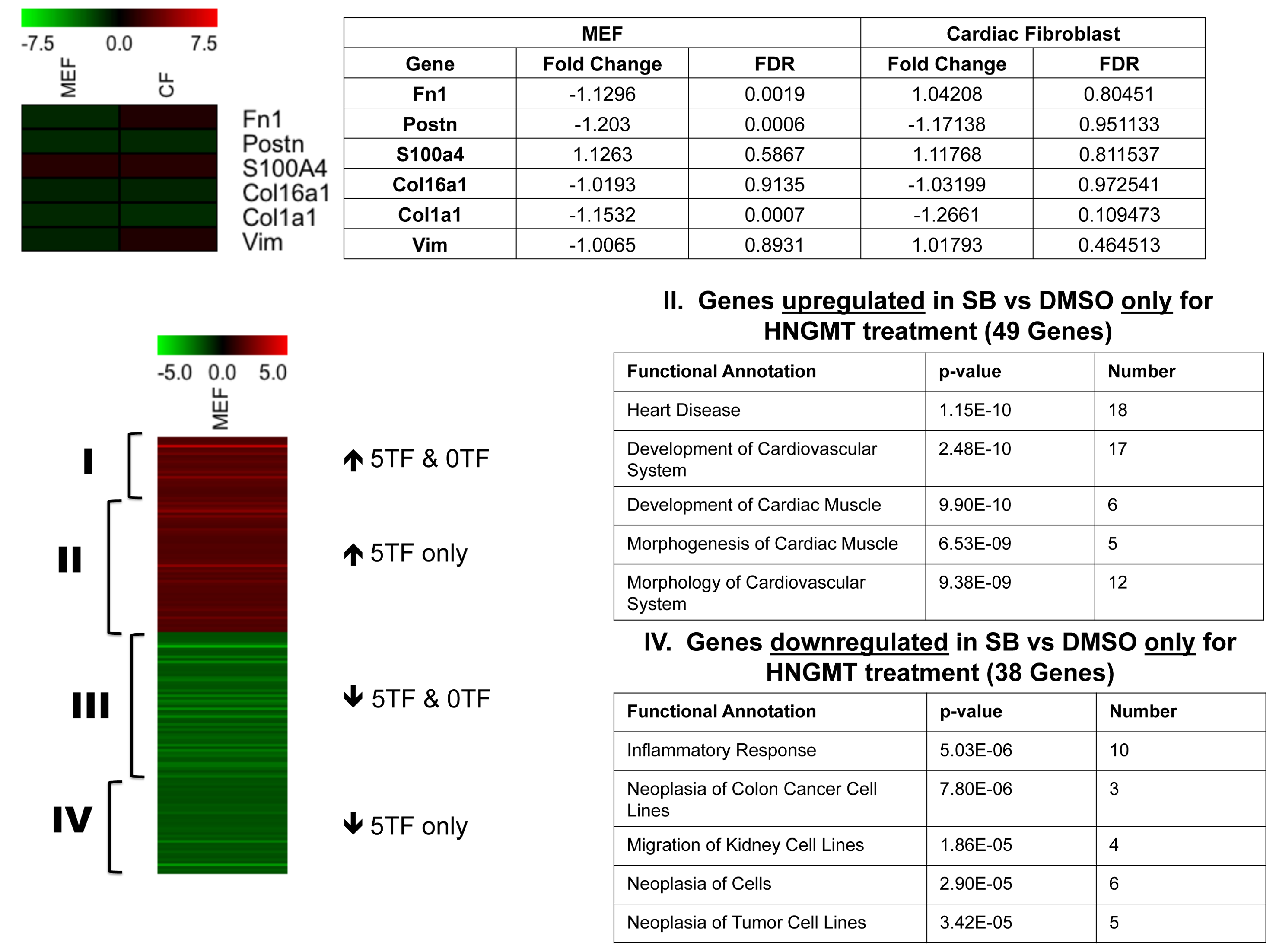

Supplement: Figure S6 — Heatmap for genes commonly associated with fibroblasts for MEFs and CFs at Day 3 post-induction in HNGMT+SB versus HNGMT+DMSO, with fold change and false discovery rate (FDR) values on the table to the right (top). Heatmap of genes up-regulated and down-regulated in MEFs for HNGMT+SB versus HNGMT+DMSO with functional annotations for the genes up-regulated or down-regulated exclusively in HNGMT+SB versus HNGMT+DMSO on the tables to the right (bottom). (TIF) [file pone.0089678.s006.tif]
