# Supplementary material for: Inhibition of TGFβ Signaling Increases Direct Conversion of Fibroblasts to Induced Cardiomyocytes
Source: PLoS One. 2014 Feb 26;9(2):e89678. doi: 10.1371/journal.pone.0089678 (PMC3935923; doi:10.1371/journal.pone.0089678)
Supplement: Table S1 — Genes up-regulated in HNGMT+SB versus HNGMT+DMSO for both MEFs and CFs at Day 3 post-induction. (DOCX) [file pone.0089678.s011.docx]

| **Gene** | **Entrez Gene Name** | **Location** | **Type** |
| --- | --- | --- | --- |
| 1600029D21Rik | RIKEN cDNA 1600029D21 | Plasma Membrane | Other |
| ANGPT2 | Angiopoietin 2 | Extracellular Space | Growth Factor |
| BMP2 | Bone Morphogenic Protein 2 | Extracellular Space | Growth Factor |
| C1S | Complement Component 1, S Subcomponent | Extracellular Space | Peptidase |
| CCL5 | Chemockine (C-C motif) Ligand 5 | Extracellular Space | Cytokine |
| Fmo1 | Flavin Containing Monooxygenase 1 | Cytoplasm | Enzyme |
| GPR116 | G Protein-Coupled Receptor 116 | Plasma Membrane | G-Protein Coupled Receptor |
| GUCY1B3 | Guanylate Cyclase 1, Soluble, Beta 3 | Cytoplasm | Enzyme |
| H3ST1 | Heparan Sulfate (glucosamine) 3-O-Sulfotransferase 1 | Cytoplasm | Enzyme |
| LBP | Lipopolysaccharide Binding Protein | Plasma Membrane | Transporter |
| NECAB1 | N-terminal EF-hand calcium binding protein 1 | Cytoplasm | Other |
| PLN | Phospholamban | Cytoplasm | Transporter |
| PPARGC1A | Peroxisome Proliferator-Activated Receptor Gamma, Coactivator 1 Alpha | Nucleus | Transcription Regulator |
| SCARA3 | Scavenger Receptor Class A, member 3 | Plasma Membrane | Transmembrane Receptor |
| SERPING1 | Serpin Peptidase Inhibitor, Clade G (C1 inhibitor), member 1 | Extracellular Space | Other |
| SLC10A6 | Solute Carrier Family 10 (sodium/bile acid cotransporter family), member 6 | Plasma Membrane | Transporter |
| SLFN5 | Schlafen Family Member 5 | Nucleus | Enzyme |
| Sp100 | Nuclear Antigen Sp100 | Nucleus | Transcription Regulator |
| TGFBR3 | Transforming Growth Factor, Beta Receptor III | Plasma Membrane | Kinase |
