# Supplementary material for: Inhibition of TGFβ Signaling Increases Direct Conversion of Fibroblasts to Induced Cardiomyocytes
Source: PLoS One. 2014 Feb 26;9(2):e89678. doi: 10.1371/journal.pone.0089678 (PMC3935923; doi:10.1371/journal.pone.0089678)
Supplement: Table S2 — Genes that are down-regulated in HNGMT+SB versus HNGMT+DMSO for both MEFs and CFs at Day 3 post-induction. (DOCX) [file pone.0089678.s012.docx]

| **Gene** | **Entrez Gene Name** | **Location** | **Type(s)** |
| --- | --- | --- | --- |
| ADAMTSL2 | ADAMT-like 2 | Unknown | Other |
| ANGPTL7 | Antiopoietin-like 7 | Extracellular Space | Other |
| CCL17 | Chemokine (C-C motif) ligand 17 | Extracellular Space | Cytokine |
| COL5A2 | Collagen, Type V, alpha 2 | Extracellular Space | Other |
| ITGB5 | Integrin, Beta 5 | Plasma Membrane | Other |
| LOX | Lysyl Oxidase | Extracellular Space | Enzyme |
| miR-181 | MicroRNA 181a-1 | Cytoplasm | microRNA |
| NREP | Neuronal Regeneration Related Protein | Cytoplasm | Other |
| NUPR1 | Nuclear Protein, Transcriptional Regulator 1 | Nucleus | Transcriptional Regulator |
| PDLIM3 | PDZ and LIM domain 3 | Plasma Membrane | Other |
| SFN | Stratifin | Cytoplasm | Other |
